# Genome Sequence of Lichtheimia ornata, an Emerging Opportunistic Mucorales Pathogen

**DOI:** 10.1128/mra.00338-23

**Published:** 2023-06-08

**Authors:** Terrance Shea, Jason T. Mohabir, Tania Kurbessoian, Brittany Berdy, James Fontaine, Andreas Gnirke, Jonathan Livny, Jason E. Stajich, Christina A. Cuomo

**Affiliations:** a Infectious Disease and Microbiome Program, Broad Institute of MIT and Harvard, Cambridge, Massachusetts, USA; b Department of Microbiology and Plant Pathology, University of California, Riverside, Riverside, California, USA; University of Maryland School of Medicine

## Abstract

Lichtheimia ornata is an emerging opportunistic Mucorales pathogen that is associated with fatal infections in immunocompromised individuals. While these environmentally acquired infections have rarely been reported to date, cases were noted in a recent analysis of coronavirus disease 2019 (COVID-19)-associated mucormycosis in India. Here, we report the annotated genome sequence of the environmental isolate CBS 291.66.

## ANNOUNCEMENT

Lichtheimia ornata is an opportunistic fungal pathogen that is an emerging cause of invasive disease. L. ornata is taxonomically classified in the order Mucorales, which includes other *Lichtheimia* and *Rhizopus* species (1). These species live in the environment as saprotrophs, and many can grow at high temperatures, which is an advantage for growth at human body temperature (1). Infections by these species can be very difficult to treat and consequently have high mortality rates (2). L. ornata was recently detected among cases of coronavirus disease 2019 (COVID-19)-associated mucormycosis (CAM) in India in 2021 (3).

The CBS 291.66 isolate of L. ornata was obtained from the Westerdijk Fungal Biodiversity Institute. The website entry for this isolate reports that it was collected from a bird dung sample in India in 1961. This isolate was cultured in malt extract-yeast extract (MEYE) agar at 20°C for >1 week until full hyphal growth reached the edges of the plate. Genomic DNA was isolated using a standard fungal cetyltrimethylammonium bromide (CTAB) extraction protocol (4) with collected fungal hyphal material, which yielded 136 ng/μL genomic DNA dissolved in water.

From genomic DNA, two Oxford Nanopore Technologies (ONT) libraries were constructed using the 1D ligation kit (catalog number SQK-LSK109), and each was loaded in a FLO-MIN106D flow cell on a GridION instrument. Base calling was performed using Guppy v6.0.7 (for SRA accession number SRR23855900) and Guppy v6.1.5 (for SRA accession number SRR23855899). Illumina sequencing was performed on the sample using two different library construction methods. One library was prepared using a standard NEBNext Ultra II protocol and sequenced on a HiSeq X Ten system with 300 cycles. The other, which was a set of four transposase enzyme-linked long-read sequencing (TELL-Seq) libraries for which reads from the same bead-bound genomic DNA molecule shared the same barcode (5), was constructed from genomic DNA depleted of <10-kb fragments using the Pacific Biosciences (PacBio) short-read eliminator (SRE) XS kit. Libraries from 0.33 and 0.67 ng of input DNA were constructed using the TELL-Seq kit (Universal Sequencing Technologies). Five- or 10-μL aliquots of the 20-μL bead-bound library were amplified by 13 to 15 PCR cycles and sequenced on a NovaSeq SP system with 300 cycles. Total coverage of 4× was generated with ONT reads (106,228 reads [read *N*_50_, 5.2 kb]), 25× with Illumina 150-bp paired-end reads, and 3,800× with TELL-Seq reads.

The TELL-Seq libraries were processed with TELL-Read analysis pipeline software (5), and the four libraries were merged into single index I1 read, R1 read, and R2 read fastq files and then converted into 10×-compatible format using the ust10x tool (https://www.universalsequencing.com/software/). Reads were assembled using Supernova v2.1.1 (6) with the following parameters: –accept-extreme-coverage, –maxreads=300000000 scaffolds >=500 bases. Gaps were closed by running three iterations of LR_Gapcloser (unversioned) (7) using ONT reads, followed by one iteration of Pilon v1.23 (8) using Illumina (NEBNext Ultra II library) reads and then three more iterations of LR_Gapcloser with ONT reads. Finally, the assembly was polished through three iterations of Pilon v1.23 using Illumina (NEBNext Ultra II library) reads. Scaffolds were aligned with the NCBI nucleotide database using BLAST v2.12.0+ (–task blastn); along with GC content and read coverage analyses, 93 scaffolds were identified as bacterial sequences and removed from the final assembly. The assembly of CBS 291.66 consists of 603 scaffolds (795 contigs), with a scaffold *N*_50_ value of 386 kb (contig *N*_50_, 151 kb) and a total length of 37.5 Mb.

The genome was annotated using a data set of 35,966 proteins from three *Lichtheimia* species (Lichtheimia corymbifera JMRC:FSU:9682 [9], Lichtheimia ramosa JMRC:FSU:6197 [10], and Lichtheimia hyalospora FSU:10163 [11]), which were used with BRAKER2 (12) to identify candidate gene structures in the assembly that had been masked using RepeatMasker v4.1 (13). Genes containing Pfam domains found in repetitive elements or overlapping tRNA/rRNA features were removed. Genes were named and numbered sequentially. Protein-coding genes were named from Pfam/TIGRFAM database (searched with HMMER) and Swiss-Prot and KEGG database (searched with BLASTp) products (14–16). A total of 13,172 genes were predicted, and BUSCO v3 (17) identified 99.3% of the mucorales_odb10 gene set.

### Data availability.

The sequence, assembly, and annotation reported here are available in GenBank under BioProject accession number PRJNA909826. The Illumina reads from the TELL-Seq libraries are available under SRA accession numbers SRR23705169 to SRR23705174. The Illumina reads from the NEBNext Ultra II library are available under SRA accession number SRR23705175. The ONT reads are available under SRA accession numbers SRR23855899 and SRR23855900. The genome assembly is available under GenBank accession number JARTCD000000000.

## References

[1] Walther G, Wagner L, Kurzai O. 2020. Outbreaks of Mucorales and the species involved. Mycopathologia 185:765–781. doi:10.1007/s11046-019-00403-1. 31734800

[2] Skiada A, Lass-Floerl C, Klimko N, Ibrahim A, Roilides E, Petrikkos G. 2018. Challenges in the diagnosis and treatment of mucormycosis. Med Mycol 56:93–101. doi:10.1093/mmy/myx101. 29538730 PMC6251532

[3] Chowdhary A, Gupta N, Wurster S, Kumar R, Mohabir JT, Tatavarthy S, Mittal V, Rani P, Barman P, Sachdeva N, Singh A, Sharma B, Jiang Y, Cuomo CA, Kontoyiannis DP. 2023. Multimodal analysis of the COVID-19-associated mucormycosis outbreak in Delhi, India indicates the convergence of clinical and environmental risk factors. Mycoses 66:515–526. doi:10.1111/myc.13578. 36790120

[4] Carter-House D, Stajich J, Unruh S, Kurbessoian T. 2020. Fungal CTAB DNA extraction. protocols.io doi:10.17504/protocols.io.bhx8j7rw.

[5] Chen Z, Pham L, Wu T-C, Mo G, Xia Y, Chang PL, Porter D, Phan T, Che H, Tran H, Bansal V, Shaffer J, Belda-Ferre P, Humphrey G, Knight R, Pevzner P, Pham S, Wang Y, Lei M. 2020. Ultralow-input single-tube linked-read library method enables short-read second-generation sequencing systems to routinely generate highly accurate and economical long-range sequencing information. Genome Res 30:898–909. doi:10.1101/gr.260380.119. 32540955 PMC7370886

[6] Weisenfeld NI, Kumar V, Shah P, Church DM, Jaffe DB. 2017. Direct determination of diploid genome sequences. Genome Res 27:757–767. doi:10.1101/gr.214874.116. 28381613 PMC5411770

[7] Xu G-C, Xu T-J, Zhu R, Zhang Y, Li S-Q, Wang H-W, Li J-T. 2019. LR_Gapcloser: a tiling path-based gap closer that uses long reads to complete genome assembly. GigaScience 8:giy157. doi:10.1093/gigascience/giy157. 30576505 PMC6324547

[8] Walker BJ, Abeel T, Shea T, Priest M, Abouelliel A, Sakthikumar S, Cuomo CA, Zeng Q, Wortman J, Young SK, Earl AM. 2014. Pilon: an integrated tool for comprehensive microbial variant detection and genome assembly improvement. PLoS One 9:e112963. doi:10.1371/journal.pone.0112963. 25409509 PMC4237348

[9] Schwartze VU, Winter S, Shelest E, Marcet-Houben M, Horn F, Wehner S, Linde J, Valiante V, Sammeth M, Riege K, Nowrousian M, Kaerger K, Jacobsen ID, Marz M, Brakhage AA, Gabaldón T, Böcker S, Voigt K. 2014. Gene expansion shapes genome architecture in the human pathogen *Lichtheimia corymbifera*: an evolutionary genomics analysis in the ancient terrestrial mucorales (Mucoromycotina). PLoS Genet 10:e1004496. doi:10.1371/journal.pgen.1004496. 25121733 PMC4133162

[10] Linde J, Schwartze V, Binder U, Lass-Flörl C, Voigt K, Horn F. 2014. *De novo* whole-genome sequence and genome annotation of *Lichtheimia ramosa*. Genome Announc 2:e00888-14. doi:10.1128/genomeA.00888-14. 25212617 PMC4161746

[11] Wang Y, Chang Y, Ortañez J, Peña JF, Carter-House D, Reynolds NK, Smith ME, Benny G, Mondo SJ, Salamov A, Lipzen A, Pangilinan J, Guo J, LaButti K, Andreopolous W, Tritt A, Keymanesh K, Yan M, Barry K, Grigoriev IV, Spatafora JW, Stajich JE. 2023. Divergent evolution of early terrestrial fungi reveals the evolution of mucormycosis pathogenicity factors. Genome Biol Evol 15:evad046. doi:10.1093/gbe/evad046. 36930540 PMC10079182

[12] Brůna T, Hoff KJ, Lomsadze A, Stanke M, Borodovsky M. 2021. BRAKER2: automatic eukaryotic genome annotation with GeneMark-EP+ and AUGUSTUS supported by a protein database. NAR Genom Bioinform 3:lqaa108. doi:10.1093/nargab/lqaa108. 33575650 PMC7787252

[13] Smit AFA, Hubley R, Green P. 2013–2015. RepeatMasker Open-4.0. https://www.repeatmasker.org/faq.html.

[14] Finn RD, Bateman A, Clements J, Coggill P, Eberhardt RY, Eddy SR, Heger A, Hetherington K, Holm L, Mistry J, Sonnhammer ELL, Tate J, Punta M. 2014. Pfam: the protein families database. Nucleic Acids Res 42:D222–D230. doi:10.1093/nar/gkt1223. 24288371 PMC3965110

[15] UniProt Consortium. 2023. UniProt: the Universal Protein Knowledgebase in 2023. Nucleic Acids Res 51:D523–D531. doi:10.1093/nar/gkac1052. 36408920 PMC9825514

[16] Kanehisa M, Sato Y, Kawashima M. 2022. KEGG mapping tools for uncovering hidden features in biological data. Protein Sci 31:47–53. doi:10.1002/pro.4172. 34423492 PMC8740838

[17] Simão FA, Waterhouse RM, Ioannidis P, Kriventseva EV, Zdobnov EM. 2015. BUSCO: assessing genome assembly and annotation completeness with single-copy orthologs. Bioinformatics 31:3210–3212. doi:10.1093/bioinformatics/btv351. 26059717

